# A Novel Synthetic Steroid of 2β,3α,5α-Trihydroxy-androst-6-one Alleviates the Loss of Rat Retinal Ganglion Cells Caused by Acute Intraocular Hypertension via Inhibiting the Inflammatory Activation of Microglia

**DOI:** 10.3390/molecules24020252

**Published:** 2019-01-11

**Authors:** Hong-Jia-Qi Sun, Dong-Dong Xue, Bing-Zheng Lu, Yuan Li, Long-Xiang Sheng, Zhu Zhu, Yu-Wei Zhou, Jing-Xia Zhang, Gan-Jian Lin, Sui-Zhen Lin, Guang-Mei Yan, Yu-Pin Chen, Wei Yin

**Affiliations:** 1 Department of Pharmacology, Zhongshan School of Medicine, Sun Yat-sen University, Guangzhou 510080, China; Elinor_Sun@outlook.com (H.-J.-Q.S.); xuedd@mail2.sysu.edu.cn (D.-D.X.); lubzh3@mail2.sysu.edu.cn (B.-Z.L.); zmd2012spring@126.com (Y.L.); shenglx@mail2.sysu.edu.cn (L.-X.S.); zhuzhucute@163.com (Z.Z.); zhouyw7@mail2.sysu.edu.cn (Y.-W.Z.); ygm@mail.sysu.edu.cn (G.-M.Y.); 2 School of Pharmaceutical Sciences, Sun Yat-sen University, 132 East Circle at University City, Guangzhou 510006, China; zhjingx@mail.sysu.edu.cn; 3 Guangzhou Cellprotek Pharmaceutical Co. Ltd., G Building F/4, 3 Lanyue Road, Science City, Guangzhou 510663, China; linganjian@cellprotek.com (G.-J.L.); linsuizhen@cellprotek.com (S.-Z.L.); 4 Department of Biochemistry, Zhongshan School of Medicine, Sun Yat-sen University, Guangzhou 510080, China

**Keywords:** 2β,3α,5α-trihydroxy-androst-6-one, lipopolysaccharide, microglial activation, nuclear factor-κ B, ischemia/reperfusion injury, acute glaucoma

## Abstract

Neuroinflammation has been well recognized as a key pathological event in acute glaucoma. The medical therapy of acute glaucoma mainly focuses on lowering intraocular pressure (IOP), while there are still scarce anti-inflammatory agents in the clinical treatment of acute glaucoma. Here we reported that β,3α,5α-trihydroxy-androst-6-one (sterone), a novel synthetic polyhydric steroid, blocked neuroinflammation mediated by microglia/macrophages and alleviated the loss of retinal ganglion cells (RGCs) caused by acute intraocular hypertension (AIH). The results showed that sterone significantly inhibited the morphological changes, the up-regulation of inflammatory biomarker ionized calcium-binding adapter molecule 1 (Iba-1), and the mRNA increase of proinflammatory tumor necrosis factor-α (TNF-α), interleukin-1β (IL-1β), and interleukin-6 (IL-6) induced by lipopolysaccharide (LPS) in BV2 microglia and RAW264.7 macrophages. Moreover, immunofluorescence and western blotting analysis revealed that sterone markedly abrogated the nuclear translocation and phosphorylation of nuclear factor-κB (NF-κB) p65 subunit. Furthermore, sterone significantly suppressed the inflammatory microglial activation and RGCs’ reduction caused by retinal ischemia/reperfusion (I/R) injury in a rat AIH model. These results suggest sterone may be a potential candidate in the treatment of acute glaucoma caused by microglial activation-mediated neuroinflammatory injury.

## 1. Introduction

Acute glaucoma is a leading cause of blindness characterized by progressive loss of retinal ganglion cells (RGCs) and axons in the optic nerves, which is mainly caused by rapid increase of intraocular pressure (IOP), i.e., acute intraocular hypertension (AIH). AIH induces the compression of blood vessels in fundus oculi and the subsequent ischemia injury of the adjacent RGCs [1]. In current treatment of acute glaucoma, IOP is the only target in medical therapy or surgery [2]. Whereas recent studies elucidate that lowering IOP alone cannot prevent RGCs death and blindness completely [3,4], these frustrating results suggest that it is an urgent need to fully excavate the pathogenetic mechanism of acute glaucoma. Currently, emerging evidence has shown that neuroinflammation is implicated with RGCs death during acute glaucoma [5,6], and a few agents with anti-inflammatory properties exert a considerable suppression effect on disease progression [7,8], implying the critical role of the inhibition of neuroinflammation in acute glaucoma treatment. In the central nervous system (CNS), resident microglia play a pivotal role in immune surveillance and signal transduction, while infiltrating macrophages from the periphery act as immune signal amplifiers. Microglia/macrophages activation in ocular tissues is considered to be a key event in acute glaucoma, which is typically characterized by the morphological change from ramified to ameboid, increased phagocytosis-related protein, such as ionized calcium-binding adapter molecule 1 (Iba-1), and excess production of pro-inflammatory molecules such as tumor necrosis factor-α (TNF-α), interleukin-1β (IL-1β) and interleukin-6 (IL-6) [9]. Numerous studies have reported that after the treatment with pro-inflammatory stimuli, such as LPS or hypoxia, microglia/macrophages with small cell bodies would shift from short and fine processes into enlargement and thickening processes, along with the development of an amoeboid shape [10,11]. Despite the role of microglia/macrophages activation being complex and elusive, a few studies have claimed that overproduction of inflammatory responses following microglia/macrophages activation may further exacerbate retinal neurodegeneration, including IOP elevation, blood-retina-barrier (BRB) deterioration, and apoptosis of RGCs [12,13,14]. Furthermore, an experimental model of glaucoma has shown that the deletion of genes related to microglial activation can prevent RGC loss [15]. Therefore, microglia/macrophages activation might be directly correlated to the pathogenesis of acute glaucoma and would be very likely developed as a promising therapeutic target for acute glaucoma. 

Nuclear factor-κB (NF-κB) as a pleiotropic transcription regulator plays a critical role in neuroinflammation-related pathogenesis via modulating microgliosis, macrophage infiltration and inflammatory cytokines production [16]. After being activated by multiple stimuli, a NF-κB subunit p65 would be released from the inhibitor κB (IκB) proteins in cytoplasm, and then translocate into the nucleus for the subsequent transcription activity [17]. In addition, efficient NF-κB-dependent transcription is often accompanied with p65 phosphorylation [18]. Hence, the nuclear translocation and phosphorylation of p65 are recognized as the molecular hallmarks of optimal NF-κB activation. NF-κB inhibitors are considered to be of therapeutic value in treating CNS disorders. Studies have also shown that inhibiting IκBα-NF-κB signals alleviates microglial inflammatory activation and neurological deficits [19,20,21]. Regarding acute glaucoma, it is reported that the elevated IOP induces NF-κB translocation and the subsequent production of IL-6 in retinal microglia [22]. Moreover, NF-κB activation could also be triggered without IOP elevation in an autoimmune glaucoma model [23]. Since NF-κB activation is generally observed in acute glaucoma, agents with an inhibitory effect on NF-κB signaling seem beneficial for glaucoma treatment by regulating neuroinflammation.

Neuroactive steroids are a category of endogenous or synthetic molecules that can physiologically regulate and protect the CNS from disorders or injuries, especially those structure-modified synthetics of endogenous steroids exhibiting longer biological half-lives and better effectiveness [24]. Emerging evidence has shown that neuroactive steroids or their metabolites could modulate ocular inflammation by targeting microglia/macrophages activation [25,26], suggesting a prospective tendency for neuroactive steroids to treat acute glaucoma. 

Herein, 2β,3α,5α-trihydroxy-androst-6-one (sterone), a polyhydric steroid designed and synthesized from the natural antioxidant 20-hydroxyecdysone [27], has been previously reported to be neuroactive by maintaining cerebellar granule neurons against glutamate-induced neurotoxicity [28]. The polyhydroxy sterone structure without cholesterol side chain might account for its effective neuroprotection. Since neuroinflammation contributes to many neurological diseases including glaucoma, it remains unknown whether sterone as a potential neuroprotectant regulates neuroinflammation within the CNS. In this study, we hypothesized that sterone engages in suppressing ocular inflammation mediated by microglia and investigated the anti-inflammation of sterone in vitro and in vivo. Our results showed that sterone significantly blocked the NF-κB signaling and suppressed the LPS-induced inflammatory activation of BV2 microglia and RAW264.7 macrophages, and markedly alleviated microglial activation and RGCs loss in a rat retinal I/R injury by AIH. Collectively, this study unravels the anti-neuroinflammatory effect of sterone, suggesting its possible applications in acute glaucoma treatment. 

## 2. Results

### 2.1. Sterone Has No Effect on the Viability of BV2 Cells and RAW264.7 Cells

**Figure 1:**
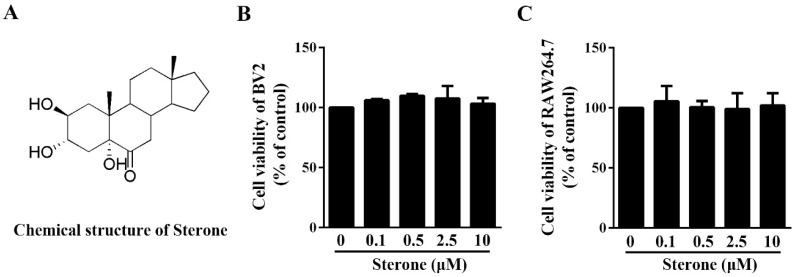
Sterone has no effect on viabilities of BV2 cells and RAW264.7 cells. Cells were incubated with the indicated concentrations of sterone for 24 h. The structure of sterone is shown in (**A**). Cell viabilities of BV2 cells (**B**) and RAW264.7 cells (**C**) were measured respectively MTT assay. Values are mean ± SD of three independent experiments.

In order to evaluate the cytotoxicity of sterone (chemical structure exhibited in Figure 1A), MTT assays were conducted in microglia or macrophages separately. As shown in Figure 1B,C, after the incubation with sterone from 0.1 μM to 10 μM for 24 h, respectively, the viabilities of BV2 cells and RAW264.7 cells were not affected by sterone. These results indicate that sterone had no detrimental effect on the survival of BV2 cells and RAW264.7 cells.

### 2.2. Sterone Inhibits LPS-Induced Inflammatory Activation of BV2 and RAW264.7 Cells.

**Figure 2:**
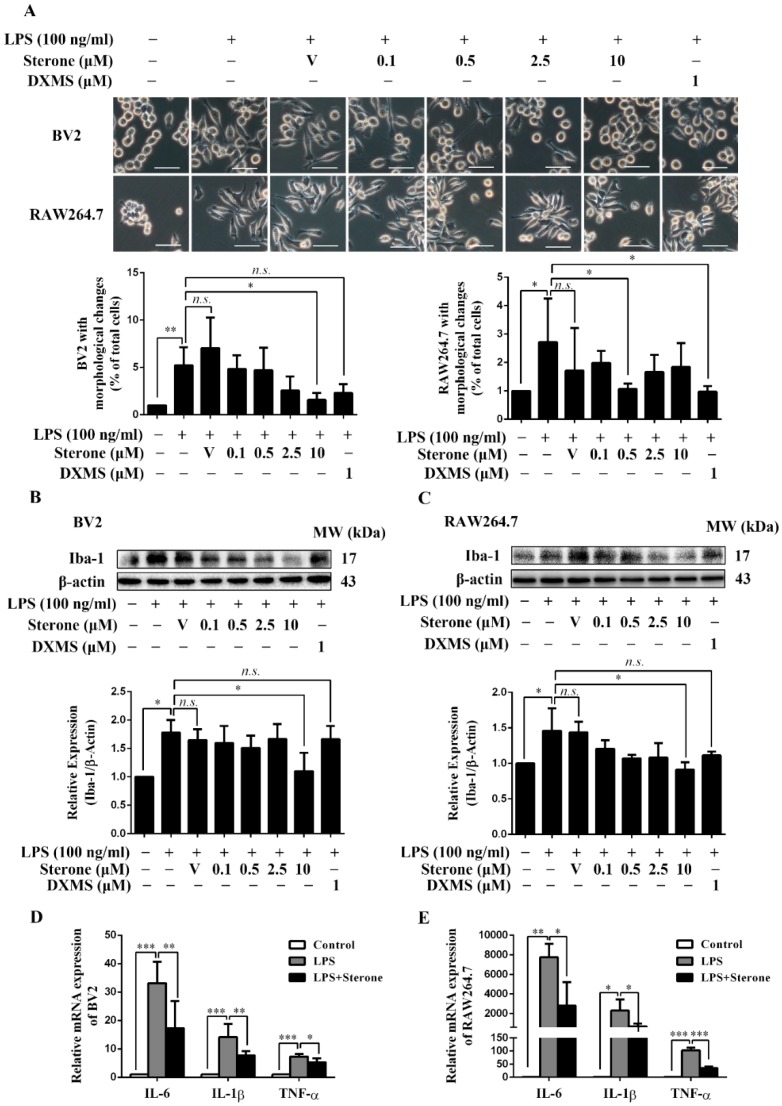
Sterone inhibited the LPS-induced inflammatory activation of BV2 and RAW264.7 cells. Cells were pre-treated with the indicated concentrations of sterone or the positive control of dexamethasone (DXMS) for 30 min before the stimulation with 100 ng/mL LPS for 6 h individually. The cellular morphological changes are presented in microphotographs and statistical graphs (**A**). Protein levels of the biomarker Iba-1 were measured using western blotting. Representative images of BV2 (**B**) and RAW264.7 cells (**C**) from more than three independent experiments are shown. The mRNA levels of IL-6, IL-1β, and TNF-α were measured using RT-qPCR respectively in BV2 (**D**) and RAW264.7 cells (**E**). V, Vehicle (HP-β-CD). Scale bars, 50 μm. Values are mean ± SD of three independent experiments. n.s, no significance. * *p* < 0.05, ** *p* < 0.01, *** *p* < 0.001.

To determine whether sterone regulates the activation of microglia/macrophages, we assayed the classical indicators of inflammatory activation including morphological changes, Iba-1 and pro-inflammatory factors in BV2 and RAW264.7 cells stimulated using LPS. Dexamethasone (DXMS) here was used as a positive control in some in vitro studies, which has been reported to strongly inhibit the LPS-induced microglia/macrophages activation and NF-κB signaling [29,30,31]. As shown in Figure 2A, the stimulation with 100 ng/mL LPS for 6 h significantly induced a shift from a short and small shape to an activated amoeboid morphology characterized by enlarged, thickened cell bodies and pseudopodia formation in both BV2 and RAW264.7 cells, while the pretreatment of 10 μM sterone in BV2 or 0.5 μM in RAW264.7 for 30 min remarkably suppressed the activation morphological changes. Then we determined the effect of sterone on the inflammatory activation biomarker of Iba-1 using western blotting. The pretreatment for 30 min of 10 μM sterone significantly attenuated the up-regulation of Iba-1 induced by 100 ng/mL LPS for 6 h in BV2 (Figure 2B) and RAW264.7 cells (Figure 2C). Consistent with the above observations, sterone blocked the mRNA level of pro-inflammatory cytokines IL-6, IL-1β, and TNF-α using RT-qPCR analysis. As shown in Figure 2D and 2E, stimulation of 100 ng/mL LPS for 6 h increased IL-6, IL-1β and TNF-α robustly in both BV2 (Figure 2D) and RAW264.7 cells (Figure 2E), which were significantly abrogated via 10 μM sterone pretreatment for 30 min. These results indicate that sterone prevents LPS-induced inflammatory activation of BV2 and RAW264.7 cells. 

### 2.3. Sterone Inhibits LPS-Induced NF-κB Activation in BV2 and RAW264.7 Cells.

**Figure 3:**
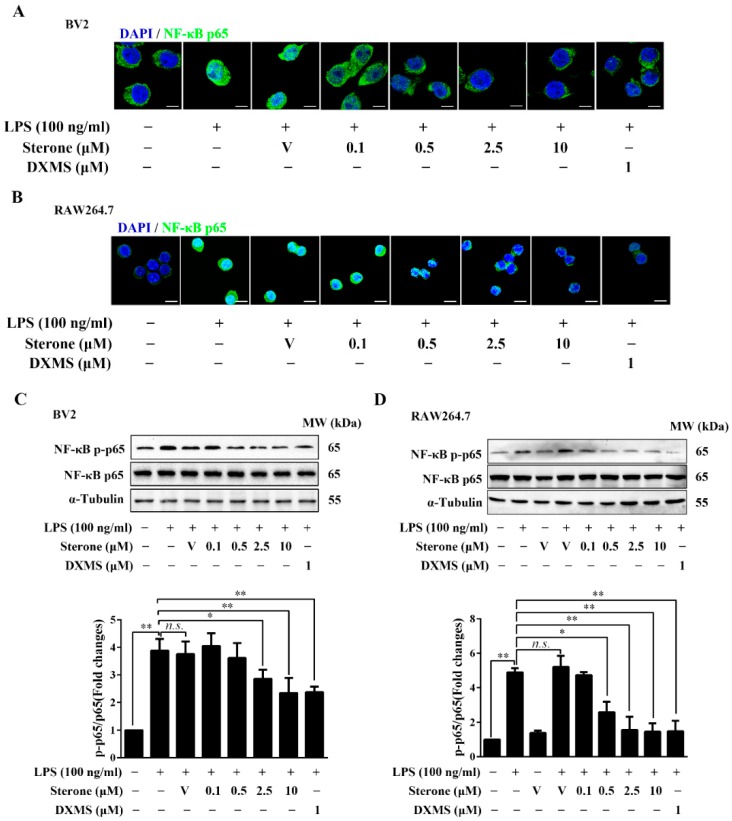
Sterone inhibited LPS-induced NF-κB activation in BV2 cells and RAW264.7 cells. Cells were pre-treated with the indicated concentrations of sterone or DXMS for 30 min before the stimulation by 100 ng/mL LPS for 30 min individually. The nuclear localization of NF-κB p65 in BV2 cells (**A**) and RAW264.7 cells (**B**) were measured using immunofluorescence stain with an NF-κB p65 antibody (Green) and nuclear stain by 4′,6-Diamidino-2-phenylindole (DAPI, blue). The phosphorylation levels of NF-κB p65 in BV2 cells (**C**) and RAW264.7 cells (**D**) were measured using western blotting. V, Vehicle (HP-β-CD). Scale bars, 10 μm. Values are mean ± SD of three independent experiments. n.s, no significance. * *p* < 0.05, ** *p* < 0.01.

Since NF-κB signaling mediates various inflammatory responses in microglia/macrophages, we then explored the effect of sterone on the nuclear translocation and phosphorylation of the p65 subunit stimulated using LPS. As shown in Figure 3, exposure to 100 ng/mL LPS for 30 min significantly induced the translocation of p65 from cytoplasm to the nucleus, while pretreatment of sterone for 30 min dose-dependently suppressed the translocation of p65 in BV2 cells (Figure 3A) and RAW264.7 cells (Figure 3B). In accordance with the results of p65 nuclear translocation, sterone in a dose-dependent manner significantly abrogated the increase of phosphorylated p65 in BV2 cells (Figure 3C) and RAW264.7 cells (Figure 3D) under 100 ng/mL LPS stimulation. These results indicate that sterone suppresses NF-κB activation induced via LPS in BV2 and RAW264.7 cells, which might account for the inhibition of sterone on the microglia/macrophages inflammatory activation. 

### 2.4. Sterone Inhibits Microglial Activation and Retinal RGCs Loss Induced via Ischemia/Reperfusion Injury in a Rat AIH Model

**Figure 4:**
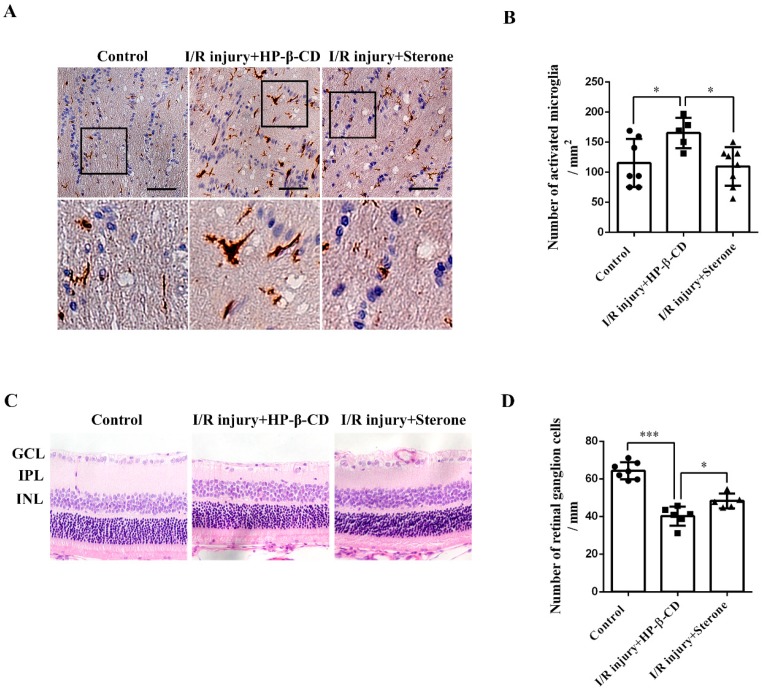
Sterone inhibited the microglial activation and retinal RGCs loss induced by ischemia/reperfusion injury in the rat AIH model. The rat AIH model was performed by retinal ischemia for 60 min, followed by sterone (80 μg/eye) treatment for 30 s and retinal reperfusion for 48 h. Microglial activation in the optic nerves was determined using Iba-1 immunohistochemistry. The immunohistochemistry results were presented in microphotographs (**A**) and statistical graphs (**B**). (**A**,**B**), n (Control) = 7, n (I/R injury+HP-β-CD) = 5, n (I/R injury+Sterone) = 8. RGCs survival was determined using light microscopic analysis. Microphotographs were obtained in (**C**) and the number of RGCs was quantified in (**D**). GCL, retinal ganglion cell layer; INL, inner nuclear layer; ONL, outer nuclear layer. (**C**,**D**), n (Control) = 7, n (I/R injury+HP-β-CD) = 6, n (I/R injury+Sterone) = 5. Scale bars, 50 μm. * *p* < 0.05, *** *p* < 0.001.

To further explore the possible anti-inflammatory and neuroprotective effect of sterone in vivo, we established a rat retinal I/R injury model of AIH by elevating IOP, and performed immunohistochemistry analyses and H&E staining respectively to determine microglial activation in the optic nerves and RGCs survival. As shown in Figure 4A,B, compared to the control group, the immunoreactivity of Iba-1 was evidently increased and Iba-1-positive microglia had a shift to ameboid morphology with enlarged, thickened cell bodies and increased pseudopodia after the retinal I/R injury, which were all remarkably abrogated by the injection of 10 mg/mL sterone. Meanwhile, compared to the control group, the number of RGCs in the ganglion cell layer (GCL) was significantly decreased after the I/R injury, while the RGC reduction was inhibited apparently in sterone-injected eyes. These results imply that sterone improved the RGCs survival by suppressing inflammatory responses mediated by microglial activation in acute glaucoma.

## 3. Discussion

Since a trend has been set in the development of neuroactive steroids as therapeutic agents for numerous CNS diseases, ocular neurological diseases including acute glaucoma may also benefit from these promising neuroprotectants, especially the structure-modified synthetic steroids. The present study showed that a synthetic compound sterone possessed anti-neuroinflammatory features by targeting the inflammatory activation of microglia/macrophages via inhibiting the NF-κB pathway. By using a rat retinal AIH model, we further confirmed that sterone protected RGCs from the microglial neuroinflammation. These findings imply that sterone may be an anti-neuroinflammatory agent candidate for acute glaucoma therapy. 

The activation of microglia/macrophages is closely correlated with the RGCs injury in acute glaucoma. Microglial activation is the most typical pathological event of neuroinflammation, which is mainly triggered and mediated by pattern recognition receptors such as Toll-like receptors (TLRs), in recognition of dangerous molecules from microbial pathogens (LPS or viral RNA) and also damaged cells (heat shock proteins or HMGB1) via topical ischemia [9]. RGCs are the ocular neurons that transmit visual information from the retina to the brain, and their progressive loss is considered a typical pathological consequence of acute glaucoma [1]. It has been reported that the inflammatory cytokines, such as TNF-α and interleukins released by activated microglia in the optic tissues, may further activate macrophage infiltration following BRB breakdown [13], and aggravate RGC apoptosis by inducing apoptosis cascades and matrix metalloproteinases elevation [5,15]. In addition, microglial activation is involved in the decrease of synapses and axonal degeneration in the optic nerve head [32,33]. In the experimental glaucoma models, treatments with minocycline, X-ray radiation or a modified erythropoietin (EPO) help preserve RGCs and ameliorate the pathological progression at least in part by inhibiting microglia/macrophages activation [32,33,34,35], suggesting microglial activation being a potential target for resisting inflammatory injury and maintaining RGC survival. In this study, we observed that sterone blocked the microglial activation possibly via the inhibition of NF-κB pathway in vitro, and sterone maintained RGCs survival possibly via the suppression of microglial activation in vivo. However, more works are required to further demonstrate sterone protects RGCs through an anti-inflammation function against inflammatory signaling-mediated microglial activation. 

As an inflammatory transcription factor, NF-κB takes a critical part in regulating various down-stream pro-inflammatory cytokines, including TNF-α and interleukins [17]. The glaucomatous retinal proteome analysis reveals that TLR/NF-κB signaling is widely activated and these inflammatory pathways may contribute to the early progression of acute glaucoma [9,34,36]. Here we reported that sterone prominently inhibited NF-κB activation by dose-dependently abrogating the nuclear translocation and phosphorylation of NF-κB p65 subunit, suggesting the role of the NF-κB pathway for sterone in suppressing microglia/macrophages activation. Tools such as siRNA for targeting NF-κB will be used to further verify the role of NF-κB on the sterone mediated anti-inflammation cascades of microglial activation in the optic nerves and RGC loss in the pathogenesis of acute glaucoma.

In our previous reports, several neuroprotective steroids exert an anti-inflammatory or antioxidant effect via inhibiting NF-κB signaling or glutamate/NMDA receptor pathways [37,38], while sterone acted as a neuroprotectant and protected the neurons from glutamate damage directly with an unknown mechanism [28]. Interestingly, some studies show that inflammatory responses, including NF-κB activation, can also be attenuated by inhibiting NMDA receptors [39,40], suggesting sterone may also exert an anti-inflammatory property via targeting NMDA receptors. Therefore, further studies will be carried out to screen and validate the specific molecular target of sterone such as the NMDA receptor.

## 4. Materials and Methods 

### 4.1. Cell Culture and Treatment

BV2 microglia and RAW264.7 macrophages were purchased from China Center for Type Culture Collection (Wuhan, China). Cells were maintained in Dulbecco’s Modified Eagle Medium (DMEM, Gibco, Carlsbad, CA, USA, 11965-118) containing 10% fetal bovine serum (FBS, Gibco, Carlsbad, CA, USA, 10099-141) and incubated in a humidified atmosphere of 5% CO_2_ and 95% air at 37 °C.

Sterone was dissolved to a concentration of 5 mg/mL stock in 20% hydroxypropyl-beta-cyclodextrin (HP-β-CD, Xi’an Deli Biology & Chemical Industry Co., Ltd, Xi’an, China, 20100309) solution. Cells were incubated with sterone for 30 min before the stimulation of LPS (100 ng/mL, Sigma, Shanghai, China) for different durations.

### 4.2. Cell Viability Analysis

Cell viability was measured using (3-(4,5-dimethylthiazolyll-2)-2,5-diphenyltetrazolium bromide) (MTT, Sigma, St. Louis, MO, USA) assay. BV2 cells and RAW264.7 cells were separately plated in cell culture plates at a density of 1.5 × 10^3^ cells/well and 2 × 10^3^ cells/well and cultured overnight. Various concentrations of sterone were added to each well and incubated for 24 h. The MTT solution (8.3 mg/mL) was then added and incubated for 4 h at 37 °C, followed by the removal of the medium from each well. Thereafter, the DMSO solution (100 μL/well) was added. After the formazan crystals had dissolved, absorbance was read at 570 nm.

### 4.3. Western Blotting Analysis

The cells were lysed in M-PER™ Mammalian Protein Extraction Reagent (Thermo Fisher Scientific, Rockford, IL, USA, 78503) supplemented with protease inhibitor cocktail (Millipore, San Diego, CA, USA, 539131-10 VL). The concentration of protein was determined using the bicinchoninic acid (BCA) protein assay (Thermo Fisher Scientific, USA, 23225). After 20 μg of total proteins in the lysates were separated on sodium dodecyl sulfate-polyacrylamide gel electrophoresis (SDS-PAGE) gels and transferred to polyvinylidene fluoride (PVDF) membranes, the western blotting analysis was performed using antibodies against Iba-1 (Abcam, Cambridge, MA, USA, ab5076), β-actin (Arigo, Hsinchu, Taiwan, ARG62346), NF-κB p65 (Santa Cruz., Dallas, TX, USA, sc-8008), Phospho-NF-kB p65 (CST, Danvers, MA, USA, 3033), and α-Tubulin (Arigo, Hsinchu, Taiwan, ARG65693). The immune complexes were visualized using an immobilon western chemilum HRP substrate (MILLIPORE, San Diego, CA, USA, WBKLS0500) according to the manufacturer’s instructions.

### 4.4. Quantitative Real-Time PCR (RT-qPCR) Analysis

Total RNA was extracted from cells using the Trizol reagent (Thermo Fisher Scientific, USA, 15596-018), according to the manufacturer’s instructions. Quantifying RNA was performed using Nanodrop 2000 (Thermo Scientific, USA). The cDNAs were synthesized from 2 μg mRNA and RT-PCR was done using SuperReal qPCR PreMix (SYBR Green, Tiangen, Beijing, China, FP202-01). Reactions were performed with 7500 Fast Real-time PCR system (Applied Biosystems, Foster City, CA, USA). The thermal cycling protocol included initial heating at 95 °C for 15 min followed by 40 cycles of 95 °C for 10 s, 62 °C for 30 s, and 72 °C for 30 s. The following primers were used for PCR: for IL-6 gene (Forward, 5′-GCCTTCTTGGGACTGATGCT-3′; Reverse, 5′-TGCCATTGCACAACTCTTTTCT-3′), for TNF-α gene (Forward, 5′-ATGGCCTCCCTCTCATCAGT-3′; Reverse, 5′-TGGTTTGCTACGACGTGGG-3′), for IL-1β gene (Forward, 5′-TGCCACCTTTTGACAGTGATG-3′; Reverse, 5′-AAGGTCCACGGGAAAGACAC-3′), and for β-actin gene (Forward, 5′-AGATCAAGATCATTGCTCCTCCT-3′; Reverse, 5′-ACGCAGCTCAGTAACAGTCC-3′).

### 4.5. Immunofluorescence Analysis

The cells were fixed in 4% paraformaldehyde for 20 min and incubated in 0.2% Triton X-100 for 15 min at room temperature. After being washed three times in phosphate-buffered saline (PBS) containing 0.1% Tween 20 (PBST), the cells were then incubated with the primary antibody against NF-κB p65 (Santa Cruz., USA, sc-8008) overnight at 4 °C in antibody diluent with background reducing components (DAKO, Santa Clara, CA, USA, S3022). The next day, the cells were washed and incubated with fluorescence-conjugated secondary antibodies (Molecular Probes, Carlsbad, CA, USA) for 1 h. Images were captured with an Inverted/Fluorescence Microscope (Olympus., Tokyo, Japan, IX71).

### 4.6. Retinal Ischemia/Reperfusion of AIH and Treatment

The 30 adult male Sprague Dawley rats (The Animal Center of Southern Medical University, Guangzhou, China) were housed under environmentally controlled conditions (12 h light–dark cycle). Rats were anesthetized with sodium pentobarbital. The right eye of each rat was dilated using 1% tropicamide before being corneal topically anesthetized with 0.5% tetracaine hydrochloride. Intraocular pressure was raised by 130 mmHg for 60 min. This was done by elevating a saline reservoir containing 0.9% NaCl and connected to a 30-gauge needle, which was placed into the anterior chamber of right eye. Retinal ischemia was confirmed by observing whitening of the fundus oculi. After ischemia for 60 min, drug treatment (80 μg/eye) were conducted to the same eye for 30 s via intravitreal injection with a microinjector connected to a 30-gauge needle. The other eye remained untreated and served as a control. After surgery, animals were corneal topically anesthetized with tetracycline cortisone eye ointment. At 48 h after I/R, the rats were anesthetized deeply with sodium pentobarbital via an intraperitoneal injection at a dose of 100 mg/kg and decapitated. Rats were excluded from analysis only if they died of failed modeling. The optic nerves were removed and fixed in modified fixative solution (modified fixative solution (glacial acetic acid:40% formaldehyde solution:saline solution:75% ethanol = 1:2:7:10 (*v*/*v*)) for 6 h before being fixed in 4% paraformaldehyde for 42 h. After fixation, retina cups were cut with a scalpel by about 2/5, leaving the intact optic nerves and the hemi-lateral retinal cups. The tissues were embedded in 62 °C paraffin before being cut into 5 mm slices along the direction of optic nerve. During the whole procedure, investigators were blinded with regard to the treatment group.

Sterone was synthesized by the Guangzhou Cellprotek Pharmaceutical Co. Ltd., China and dissolved to a concentration of 10 mg/mL using 20% HP-β-CD (Xi’an Deli Biology & Chemical Industry Co., Ltd, Xi’an, China, 20100309).

### 4.7. Immunohistochemistry Analysis and Hematoxylin Eosin (H&E) Staining

The retina slices were deparaffinized and hydrated before subsequent experiments.

For immunohistochemistry analysis, a HRP/DAB Detection Kit (Abcam, USA, ab80436) was used according to the manufacturer’s instructions. Briefly, the retina slices were subjected to antigen retrieval with a 3% H_2_O_2_ solution, and after being washed in PBS, the slices were incubated in an indicated primary antibody against Iba-1 (Wako, Osaka, Japan, 017-19741) overnight at 4 °C in antibody diluent with background reducing components (DAKO, USA, S3022). The next day, after being washed in PBS, the slices were subjected to an HRP-conjugated secondary antibody and then DAB substrate-chromogen mix was applied to the slices. Finally, hematoxylin counterstaining was performed.

For H&E staining, a Hematoxylin Eosin (H&E) Staining Kit (Nanjing Jiancheng Technology Ltd., Nanjing, China) was used according to the manufacturer’s instructions. Briefly, the retina slices were stained using hematoxylin for 5 min first and by eosin for 2 min afterwards. After being washed by distilled water, the slices were dehydrated and mounted by neutral resins. 

Finally, images from the above experiments were captured with a Nikon ECLIPSE Ti Inverted Microscope (Tokyo, Japan). To quantify the optical intensity of Iba-1, the number of RGCs, and the pixels of ganglion cell layer, these images were analyzed using Image-Pro Plus 6.0 software (Bethesda, Rockville, MD, USA) by a third researcher.

### 4.8. Statistical Analysis

All data were analyzed with GraphPad Prism 6 software (San Diego, CA, USA) and are presented as the mean ± SD. Post-hoc multiple comparisons between the groups was performed using one-way ANOVA followed by Dunnett’s multiple comparisons test. *p*-Values less than 0.05 were considered statistically significant.

## 5. Conclusions

In conclusion, this present study illuminates for the first time that sterone as a novel neuroactive steroid inhibits the inflammatory activation of microglia and protects RGCs from glaucomatous insult, which may be mechanistically correlated with the blockade of NF-κB signaling. Consequently, our work provides a new possibility that sterone is a promising agent for pharmaceutical research in acute glaucoma therapy.

## 6. Patents

J.-X.Z. and S.-Z.L. possess a patent 2β,3α,5α-trihydroxy-androst-6-one and preparation methods and use thereof, which have been issued as the PCT/CN2014/074318 application in China, United States, Europe, Japan, Korea, Russia, Singapore, Australia, and Canada.
